# Supplementary material for: Skillful subseasonal Indian Ocean marine heatwave forecasts using a neural network
Source: Environ Data Sci. 2026 Feb 24;5:e6. doi: 10.1017/eds.2026.10033 (PMC12980662; doi:10.1017/eds.2026.10033)
Supplement: Howard et al. supplementary material [file S2634460226100338sup001.pdf]

Appendix A: Ablation Study

1. METHODS

Three different NN architectures were tested after preliminary hyperparameter tuning: a standard UNet, a U-Net with skip connections removed, and a NN with only convolutional layers. These model architectures were chosen to explore the relative importance of large-scale and small-scale features in the final forecast skill. A U-Net captures both large and small scale features. A U-Net with no skip connections can use only larger scale patterns for prediction. Last, a model with only convolutional layers can use only relatively local information for predictions. Each was evaluated based on its predictive performance on validation data withheld during the training process. The hyperparameters were kept constant to the extent applicable across the three architectures. Each network has 8 hidden layers, a 3x3 convolutional kernel size with 16 feature maps in the first hidden layer.

2. RESULTS

After training all three models, forecast metrics were evaluated for each on the test dataset: CRPS, RMSE (using the predicted mean SST), and normalized RMSE. The results are shown in table S1. All three models have similar validation RMSE values. The U-Net notably outperforms both other models in terms of CRPS and normalized RMSE. This indicates that the U-Net is better able to accurately and reliably characterize uncertainty in its predictions than the other two models. On S2S timescales, this is a key feature of any skillful forecast, making the U-Net the best choice.

	U-NET	NoSkip	CNN
Val CRPS	0.2770	0.2800	0.2822
Val RMSE (K)	0.5159	0.5156	0.5169
Val RMSE Normalized	1.0661	1.1170	1.1162

Table S1. Model performance comparison
